# Interferon-stimulated Viperin impairs Treg function in autoimmune thrombocytopenia

**DOI:** 10.1186/s12964-025-02511-6

**Published:** 2025-11-20

**Authors:** Tengda Li, Xiang Li, He Huang, Peng Liu, Zhifa Shen, Chang Xue

**Affiliations:** 1 Key Laboratory of Laboratory Medicine, Ministry of Education, School of Laboratory Medicine and Life Sciences, Wenzhou Medical University, Wenzhou, Zhejiang 325035 China; 2 Department of Chemistry, Fudan University, Shanghai, 200438 China; 3 Department of Hematology, The Second Affiliated Hospital and Yuying Children’s Hospital of Wenzhou Medical University, Wenzhou, Zhejiang China; 4 Department of Blood Transfusion, No.971 Hospital of the PLA Navy, Qingdao, China

**Keywords:** Primary immune thrombocytopenia, Regulatory T cell, Interferon-Stimulated gene, Viperin, Single-Cell analysis

## Abstract

Primary immune thrombocytopenia (ITP) is an autoimmune disorder driven by dysfunctional regulatory T cells (Tregs) that mediate platelet destruction. Here, we show that Tregs from ITP patients undergo a profound shift in subset composition and transcriptional states, with expansion of ANXA1^high^ and IKZF2^high^ populations and aberrant interferon signaling. Single-cell transcriptomic and functional analyses revealed that in normal controls, immature-like Tregs predominantly exhibit a FOXP3^high^/CCR6^high^ phenotype, whereas in chronic ITP they adopt an ANXA1^high^ state enriched for interferon-stimulated gene (ISG)^high^ subclusters. Elevated ISG scores in chronic ITP Tregs mark a pathological transition, with RSAD2 (Viperin) emerging as a key regulator. Viperin overexpression impaired Treg suppressive function and promoted Th1-skewed activation in conventional T cells. Mechanistically, ELF1 directly binds the RSAD2 promoter and activates its transcription via increased H3K4Me3 deposition. These findings identify the ELF1-Viperin axis as the driver of Treg dysfunction in ITP and a potential target for therapeutic intervention.

**Supplementary Information:**

The online version contains supplementary material available at 10.1186/s12964-025-02511-6.

## Introduction

Primary immune thrombocytopenia (ITP) is an autoimmune hematological disorder characterized by reduced platelet counts [1]. The mechanisms underlying ITP remain unclear, but experts believe that immune system dysregulation is a critical factor driving disease onset and progression [1, 2]. Due to the lack of understanding of the disease’s etiology, current clinical treatments, such as intravenous administration of glucocorticoids, often show limited efficacy [1, 2]. Causal treatments targeting the disease’s underlying mechanisms have not yet been developed. Investigating the immunological pathogenic mechanisms of ITP in depth may provide novel therapeutic targets for effective treatment.

Regulatory T cells (Tregs) play a central role in immunosuppression, preventing autoimmune diseases by controlling the excessive proliferation of inflammatory cells, such as conventional T cells [3]. Studies have shown that Treg dysfunction or reduced numbers can lead to severe autoimmune responses, which in extreme cases may be fatal [3, 4]. Tregs maintain their immunosuppressive functions through the stability of their core transcriptional regulatory networks and a well-established repertoire of immune molecules [3, 5]. The production of effector or inflammatory molecules by Tregs often indicates a partial loss of their original function [5]. In ITP patients, restoring Treg functionality through therapeutic interventions has been shown to help restore platelet counts and slow disease progression [6, 7]. Additionally, in vivo experiments demonstrate that enhancing Treg-mediated immune regulation can reduce macrophage-mediated platelet phagocytosis [8], underscoring the critical role of Tregs in ITP pathogenesis. With advances in technology, single-cell sequencing has become a preferred tool for researchers [9]. Analyzing Treg functionality and associated abnormalities at the single-cell level in ITP patients will enable the identification of Treg cell heterogeneity and a deeper understanding of their roles in ITP pathogenesis. In this study, we integrated single-cell RNA sequencing (scRNA-seq) with experimental validation to identify key molecular drivers underlying the impaired immunosuppressive function of Treg cells in ITP patients. Our findings will provide novel insights into the pathogenesis of ITP and offer potential therapeutic targets for improving clinical diagnosis and treatment.

## Materials and methods

### The information of patients

Peripheral blood samples were collected from 12 normal control (NC), 14 newly diagnosed ITP patients (N_ITP), and 14 chronic ITP patients (C_ITP). Among these, 2 N_ITP and 2 C_ITP samples were used for single-cell sequencing analysis, while the remaining samples were used for experimental validation. The average age of the NC group was 51.2 years, the average age of the N_ITP group was 50.3 years, and the average age of the C_ITP group was 51.4 years. The male-to-female ratio in each group was 1:1. NC refers to individuals with normal blood function indicators. N_ITP refers to patients diagnosed within 3 months, while C_ITP refers to patients with sustained platelet reduction for more than 12 months. The age, sex, duration of disease in chronic patients, serum C-reactive protein levels, medications, and the volume of blood collected for each patient were detailed in Table S1. In accordance with the Declaration of Helsinki, this research was approved by the ethics committee of the Second Affiliated Hospital of Wenzhou Medical University (Approval No.: 2024-K-224-01), and all participants provided written informed consent upon enrollment.

### Sample preparation

Red blood cells in the blood samples were lysed using a red blood cell lysis buffer, followed by centrifugation to remove the supernatant. The cell pellet was washed twice with PBS containing 2% FBS to ensure the removal of residual debris. The cells were then resuspended in 100 *µ*L of PBS supplemented with 2% FBS. According to the manufacturer’s instructions, antibodies specific for CD25 (BioLegend, 985812), CD4 (BioLegend, 980808), and CD127 (BioLegend, 986002) were added at the recommended concentrations. Tregs, defined as CD4⁺CD25⁺CD127^low^ cells, were subsequently isolated through flow cytometric sorting. Sorted cells with a purity greater than 90% were used for downstream analyses. Cells allocated for real-time reverse transcription quantitative polymerase chain reaction (RT-qPCR), western blotting, and Treg suppression assays were processed as described in subsequent sections. For scRNA-seq, fluorescence-activated cell sorting (FACS)-sorted Tregs were obtained from N_ITP patients (one male and one female, yielding 8,141 and 11,916 cells, respectively) and C_ITP patients (one female and one male, yielding 8,197 and 4,672 cells, respectively). These four patient-derived samples were not included in subsequent validation experiments. Single-cell libraries were prepared using the 10x Genomics Chromium Single Cell 3′ protocol with the Chromium Next GEM Single Cell 3′ Kit (v3.1 or v4) and sequenced on an Illumina NovaSeq X Plus platform.

For overexpression experiments, CD4⁺CD25⁺CD127^dim/^⁻Treg cells were isolated from the peripheral blood of normal donors using magnetic bead-based cell sorting (Miltenyi Biotec, 130-094-775). Cells with a post-sorting purity greater than 90% were confirmed and subsequently used for downstream analyses. If the purity did not exceed 90%, the cells were further quickly enriched by FACS to achieve a purity above 90% prior to downstream experiments.

### Data preprocessing for scRNA-seq analysis

For the Treg single-cell sequencing results used in this study, the sequencing data for N_ITP and C_ITP were generated by our research team, while the normal control single-cell sequencing data was sourced from GSE175604. The single-cell sequencing analysis began with processing raw FASTQ files using Cell Ranger for read alignment, barcode assignment, and feature quantification. The output data, including gene-cell matrices, were then imported into Seurat for downstream analysis. Key steps included data normalization, dimensionality reduction, and clustering, followed by generating uniform manifold approximation and projection (UMAP) plots to visualize cell populations and explore transcriptional heterogeneity. We selected *CD4*^+^ and *IL2RA*^+^ cells (Tregs) for further analysis.

The chromatin immunoprecipitation sequencing (ChIP-seq) data were obtained from GSE40684. We converted the text files into BED format and further analyzed them using the integrative genomics viewer (IGV) to examine the binding of the transcription factors within specific regions of the target gene.

### Integrated functional analysis of scRNA-seq data

Principal component analysis (PCA): After standardizing the expression matrix and filtering low-quality cells/genes, PCA was performed to identify major sources of variance across samples. The results were visualized using 3D scatter plots to highlight differences between control and patient groups.

Gene set variation analysis (GSVA): GSVA was conducted using predefined gene sets from literature or pathway databases. Enrichment scores were calculated to assess pathway activity across Treg subsets and tumor samples.

Gene ontology (GO) pathway analysis: Differentially expressed genes were identified between tissues, followed by GO enrichment analysis using tools like STRING. Significant pathways were visualized based on adjusted p-values.

Single-cell velocity (scVelo) analysis: We used BAM files containing both spliced and unspliced RNA as input, followed by quality control and normalization. A k-nearest neighbor (k-NN) graph was constructed to estimate cell-to-cell connectivity, and dimensionality reduction was applied. RNA velocity was computed using the scv.tl.velocity() function by comparing spliced/unspliced RNA ratios, predicting future transcriptional states. Velocities were projected onto UMAP to visualize dynamic transcriptional changes and infer differentiation trajectories. We also calculated velocity length (indicating speed) and velocity confidence (reflecting consistency across cells).

Monocle and python implementation of Single-Cell rEgulatory Network Inference and Clustering (pySCENIC) analysis: Monocle was used to reconstruct pseudotime trajectories and identify genes with temporal dynamics during cell differentiation. pySCENIC analysis involved selecting highly variable genes as the focus of analysis, followed by the construction of a gene co-expression network to identify potential transcription factor (TF) regulatory modules. Motif analysis of TF binding sites, combined with DNA sequence information, was used to validate the relationship between TFs and their target genes, further refining the regulatory network(as shown in Figure S2C). By calculating the activity of TF regulons, we assessed the level of activity of specific TF clusters in different cells. The resulting data were visualized using heatmaps, scatter plots, and other methods for analysis.

### Flow cytometry staining and enzyme-linked immunosorbent assay (ELISA)

For surface marker staining, cells were incubated with fluorochrome-conjugated antibodies as described above. For intracellular forkhead box P3 (FOXP3) staining, cells were first fixed and permeabilized using a permeabilization buffer according to the manufacturer’s instructions, followed by staining with anti-human FOXP3 antibody (BioLegend, 320107). Data were acquired on a flow cytometer and analyzed using FlowJo software. The flow cytometer used for analysis and cell sorting in this study was BD FACSAria™ III system (BD Biosciences) equipped with four lasers. The fluorochromes applied included FITC, PE, and PE-Cy7.

To assess cytokine secretion by Tregs, we performed ELISA assays to quantify interferon gamma (IFN-γ) (ab100537), transforming growth factor beta 1 (TGF-β1) (ab100647), interleukin-10 (IL-10) (ab185986), tumor necrosis factor alpha (TNF-α) (ab181421), and interleukin-17 A (IL-17 A) (ab216167) (all from Abcam). Purified Tregs were stimulated with anti-CD3/CD28 antibodies and cultured for 24–48 h, after which culture supernatants were collected and clarified by low-speed centrifugation to remove cell debris. For each cytokine, a 7-point serial dilution of the provided recombinant standard (including blank) was prepared to generate a standard curve, and appropriate sample dilutions were applied to ensure measurements fell within the linear range. IL-10, TNF-α, and IL-17 A were measured using SimpleStep ELISA kits (as shown in Figure S4B), in which samples and antibody cocktail were co-incubated for 1 h, followed by a single wash, addition of 3,3′,5,5′-tetramethylbenzidine (TMB) substrate for 10 min, termination of the reaction, and measurement at 450 nm, with a total assay time of approximately 90 min. IFN-γ and TGF-β1 were measured using conventional sandwich ELISA, with sequential addition of samples, biotinylated detection antibody, HRP-streptavidin, TMB substrate, and stop solution prior to reading at 450 nm, with a total assay time of 3–4 h. Notably, as TGF-β1 is predominantly present in a latent complex in biological samples, an acid/alkali activation step was performed according to the manufacturer’s instructions to release the active form prior to measurement. All standards and samples were assayed in duplicate, and standard curves were fitted using a logistic regression model to calculate cytokine concentrations, which are reported as pg/mL.

### RT-qPCR and western blot

We first lysed the cells using Trizol to extract total RNA, ensuring an OD260/OD280 ratio greater than 1.8. The extracted RNA was then reverse-transcribed into cDNA. Subsequently, primers, reaction mixture, enzyme mix, DEPC-treated water, Oligo(dT)18, and the cDNA template were added to prepare the reaction system for analysis. qPCR analyses were performed on the Applied Biosystems™ 7500 Real-Time PCR System (Thermo Fisher Scientific). The relative mRNA levels were calculated based on the corresponding formula (results as shown in Figure S3). The primer information used in this study was provided in Table S2.

Cells were lysed using RIPA buffer supplemented with protease and phosphatase inhibitors. Equal amounts of protein were separated by SDS-PAGE and transferred onto PVDF membranes. Membranes were blocked with 5% non-fat milk in TBST and incubated overnight at 4 °C with primary antibodies. After washing, membranes were incubated with HRP-conjugated secondary antibodies and visualized using enhanced chemiluminescence. The primary antibodies used in our experiments included anti-GAPDH (Abcam, ab9485), anti-Viperin (Abcam, ab208286), anti-ELF1 (Abcam, ab229563), and Goat Anti-Rabbit IgG H&L (HRP) (Abcam, ab205718).

### Treg suppressive assay

Human peripheral conventional CD4⁺T cells were isolated and resuspended in serum-free PBS at approximately 5 × 10^6^ cells/mL. Cells were then incubated with pre-diluted CFSE working solution (1–2 *µ*M, prepared from a 5 mM CFSE stock in DMSO) for 8 min at room temperature in the dark with gentle mixing. The staining reaction was immediately quenched by adding 10 volumes of complete medium containing 10% FBS, followed by centrifugation at 300–400 g for 5 min to remove the supernatant. Cells were washed 2–3 times with 10 volumes of complete medium, resuspended in fresh complete medium, and rested at 37 °C for 10–15 min before subsequent culture.

To assess the suppressive function of Treg cells, CFSE-labeled conventional CD4⁺T cells were co-cultured with Tregs at defined ratios in wells pre-coated with anti-CD3 (Biolegend, 317325) and anti-CD28 (Biolegend, 302944) antibodies. After three days of incubation, cells were harvested for analysis.

### Gene’s overexpression in Tregs

Prior to transfection, cells were pre-activated with anti-CD3/CD28 magnetic beads and recombinant human IL-2 (Biolegend, 589106, 300 IU/mL) for 24 h. Subsequently, 1–2 *µ*g of overexpression or empty control plasmid was electroporated into 1 × 10⁶ cells using a Nucleofector device with the P3 Primary Cell Kit (Bioscience, V4XP-3032) and a pre-optimized electroporation program. Immediately after electroporation, cells were transferred into pre-warmed complete RPMI-1640 medium supplemented with 10% FBS and IL-2, and incubated. To ensure cross-assay consistency, fixed time points were used throughout: total RNA was collected 24 h post-electroporation, and proteins were analyzed at 48 h. Where indicated, transgene-positive Tregs were FACS-enriched at 24–36 h post-electroporation. IL-2 (300 IU/mL) was maintained in all cultures.

### Structural modeling of TF-DNA interaction

We first retrieved the protein sequence of the TF from the AlphaFold Protein Structure Database and obtained the target gene sequence from the NCBI database. The TF and target DNA sequence were then used as input for AlphaFold3 to predict the protein-DNA complex structure. The resulting model was analyzed using PyMOL to examine the interaction interface, including key amino acid residues and DNA bases. Site-directed mutagenesis was performed in silico on the identified critical amino acids, followed by re-prediction and structural analysis. Changes in the interaction between the TF and DNA before and after mutation were evaluated to assess the impact of specific residues on DNA binding.

### Statistical analysis and software applications

For comparisons between two groups of continuous variables, a *t*-test was applied if the data met the assumptions for parametric testing. Otherwise, non-parametric tests were used. The significance level was set at *P* < 0.05. The scVelo analysis for single-cell sequencing was performed on the CentOS Stream 9 system, while all other analyses were conducted in R version 4.4.1. Statistical graphs were generated using GraphPad Prism.

## Results

### Landscape of Treg subset characteristics revealed enrichment of *ANXA1*^*high*^ and *IKZF2*^*high*^ clusters in ITP Patients and *FOXP3*^*high*^, *CCR6*^*high*^, *CCR7*^*high*^ clusters in normal controls

**Fig. 1:**
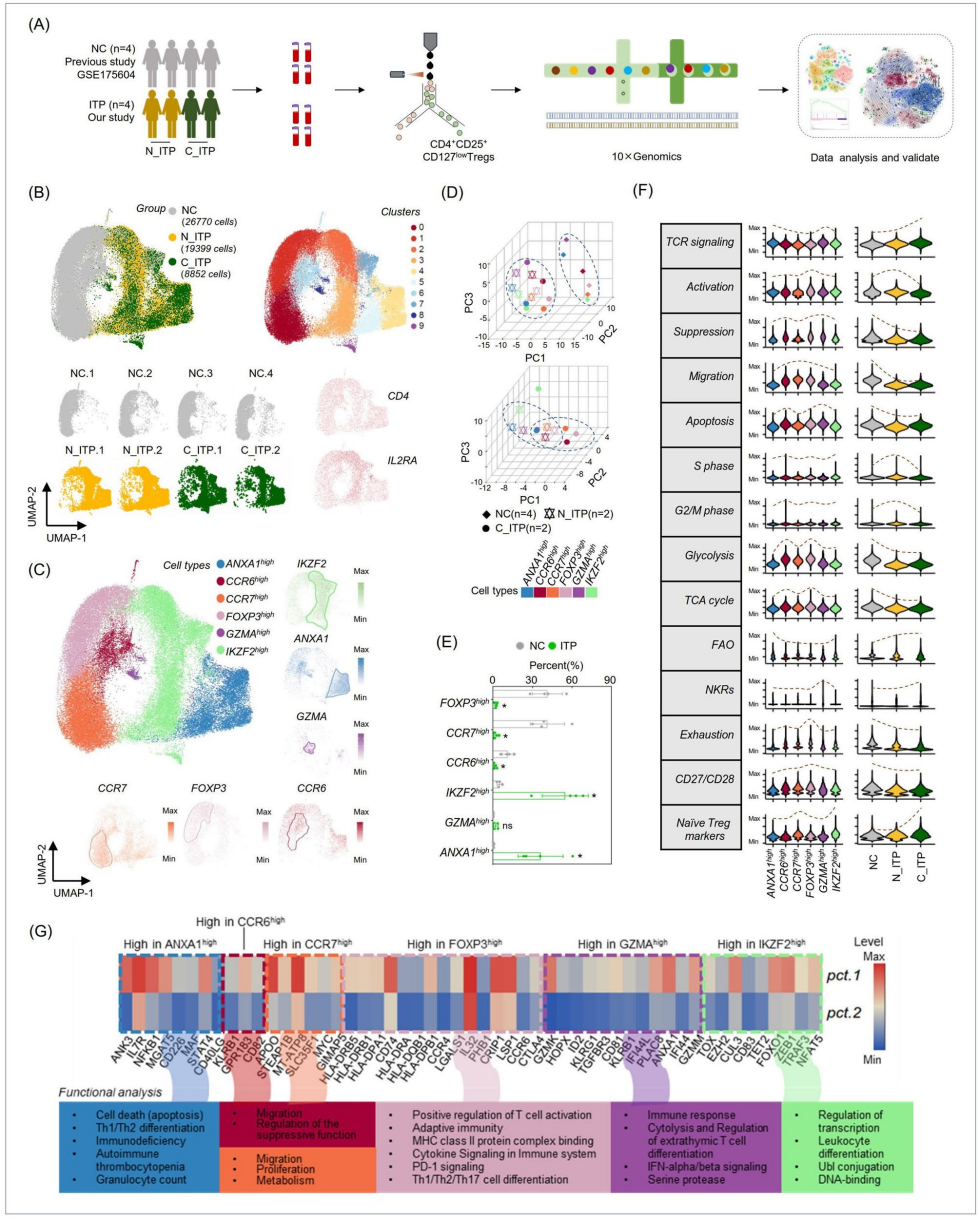
A comprehensive single-cell RNA sequence (scRNA-seq) analysis of the heterogeneity of CD4⁺CD25⁺regulatory T cells (Tregs) in the peripheral blood of normal individuals (NC), newly diagnosed immune thrombocytopenia (N_ITP) patients, and chronic ITP (C_ITP) patients. **A**A workflow diagram illustrating the study design and experimental procedures. **B** Integration and clustering analysis of scRNA-seq data of CD4⁺CD25⁺Tregs from different origins. **C** Annotation of distinct Treg subpopulations based on gene expression differences and literature references. **D** Principal component analysis (PCA) to explore the overall differences among Treg subpopulations from different sources. **E** Comparison of the proportional distribution of Treg subpopulations in the peripheral blood of NC, ITP patients. **F** Scoring analysis of key signaling pathways, including TCR signaling, activation, and suppression, in different Treg subpopulations to identify their dynamic changes. **G** Heatmap visualization of significantly upregulated key immune molecules in distinct Treg subpopulations, along with pathway enrichment analysis of the genes showing significant upregulation to uncover their biological functions. *P<0.05; ns, no significance.

We conducted single-cell sequencing analysis of Tregs from the peripheral blood of four NC and four ITP patients (two N_ITP and two C_ITP patients). The analytical workflow was shown in Fig. 1A. The samples were integrated and subjected to UMAP clustering analysis, and the cells which both expressed *IL2RA* (the gene encoding CD25) and CD4 were selected for further analysis (Fig. 1B). We called those cells as CD4⁺CD25⁺ Tregs or Tregs as below. The results revealed that Tregs from normal individuals were predominantly located in clusters 0, 1, and 6 (overall on the left), while Tregs from ITP patients clustered mainly in groups 2–5 and 7–9 (overall on the right), as shown in Fig. 1B. Based on literature and gene expression patterns [3, 10–17], we annotated the cells into six Treg subpopulations: Annexin A1(*ANXA1*)^high^, C-C Motif Chemokine Receptor (*CCR*) 6^high^, Ikaros Family Zinc Finger 2 (*IKZF2*)^high^, Granzyme A (*GZMA*)^high^, *CCR7*^high^, and *FOXP3*^high^ (Fig. 1C). These six marker genes, including *ANXA1*,* CCR6*, and *IKZF2*, play critical immunological roles in Tregs [3, 10, 11, 14, 18].

We then performed PCA on these six Treg subpopulations and found that Tregs from NC could be clearly distinguished from those of ITP patients (Fig. 1D, top panel). Furthermore, Tregs from N_ITP and C_ITP patients were also separable (Fig. 1D, bottom panel), indicating distinct characteristics among Tregs from NC, N_ITP, and C_ITP sources. Comparatively, we observed a significant increase in the proportions of *ANXA1*^*high*^ and *IKZF2*^*high*^ subpopulations in ITP patients (Fig. 1E). In contrast, the proportions of *FOXP3*^*high*^, *CCR6*^*high*^, and *CCR7*^*high*^ subpopulations were significantly reduced in Treg from ITP patients (Fig. 1E). These results suggest that the major functional Treg subpopulations are altered in ITP patients compared to normal individuals. It was important to note that, due to the inherent limitations of single-cell transcriptomic analysis, clustering was performed based on UMAP rather than marker-specific expression. As a result, *FOXP3*^*high*^ cells could not be isolated as an independent cluster. Therefore, the designation of *FOXP3*^*high*^ was based on the average *FOXP3* expression at the cluster level. Accordingly, Fig. 1E reflects only a trend indicating a reduction of *FOXP3*^*high*^ Treg cells in ITP patients, but does not imply that these represent the absolute number of *FOXP3*^*high*^ Tregs present in the samples.

Next, we assessed key pathways, including the T-cell receptor (TCR) signaling pathway, activation, and suppression pathways, across Treg subpopulations, following literature-based scoring methods [14, 19]. The results showed that from NC to N_ITP to C_ITP, pathways with progressively increasing scores included TCR signaling, fatty acid oxidation (FAO) signaling, natural killer receptor (NKR) signaling (including killer cell lectin like receptor B1 (*KLRB1*)), and naïve Treg markers (Fig. 1F). The upregulation of naive Treg markers in ITP patients suggests an increased presence of undifferentiated or early-stage Treg cells, potentially indicating impaired maturation or functional deficiency. Concurrently, elevated TCR signaling implies that these cells have received antigenic stimulation and are in an early phase of activation, but have not yet transitioned into effector or memory Tregs. This coexistence may reflect an intermediate state of “partial activation without full differentiation”, suggesting disrupted activation or functional dysregulation of Tregs in ITP. Specifically in C_ITP patients, the simultaneous expression of naive signatures and heightened TCR signaling may represent a dysregulated Treg compartment under prolonged autoantigen stimulation. In contrast, pathways with progressively decreasing scores included suppression, migration, glycolysis, apoptosis, tricarboxylic acid (TCA) cycle, exhaustion, and the CD27/CD28 signaling pathway (Fig. 1F). The pathways’ scores of activation, S phase, and G2/M phase were highest in Tregs from N_ITP patients (Fig. 1F). In the *IKZF2*^*high*^ subpopulation, the naïve Treg markers showed the highest scores (Fig. 1F), consistent with previous literature [14]. Meanwhile, the *FOXP3*^*high*^ subpopulation exhibited a marked increase in CD27/CD28 signaling (Fig. 1F).

Pathway enrichment analysis of genes significantly upregulated in each Treg subpopulation revealed distinct functional differences. In ITP patients, the dominant Treg subpopulations (*ANXA1*^*high*^, *IKZF2*^*high*^, and *GZMA*^*high*^) were primarily enriched in pathways such as Th1/Th2 differentiation, immune deficiency pathways, autoimmune thrombocytopenia pathways, IFN-alpha/beta signaling, and regulation of transcriptional (Fig. 1G). In contrast, the dominant Treg subpopulations in normal individuals (*FOXP3*^*high*^, *CCR6*^*high*^, and *CCR7*^*high*^) were enriched in pathways related to migration, regulation of the suppressive function, proliferation, positive regulation of T cell activation, adaptive immunity, MHC class II protein complex binding, and cytokine signaling in immune system (Fig. 1G). In summary, the pathway analysis highlights that Tregs from ITP patients exhibit significant immune deficiencies and abnormalities in the interferon (IFN)-alpha/beta signaling pathway, whereas Tregs from normal individuals maintain prominent signaling pathways related to activation, migration, and suppressive functions.

### *ANXA1*^*high*^ Tregs exhibited self-renewal and differentiated into *FOXP3*^*high*^ and *CCR6*^*high*^ subsets, with ISGs playing a key role in *ANXA1*^*high*^ Tregs’ maintenance

**Fig. 2:**
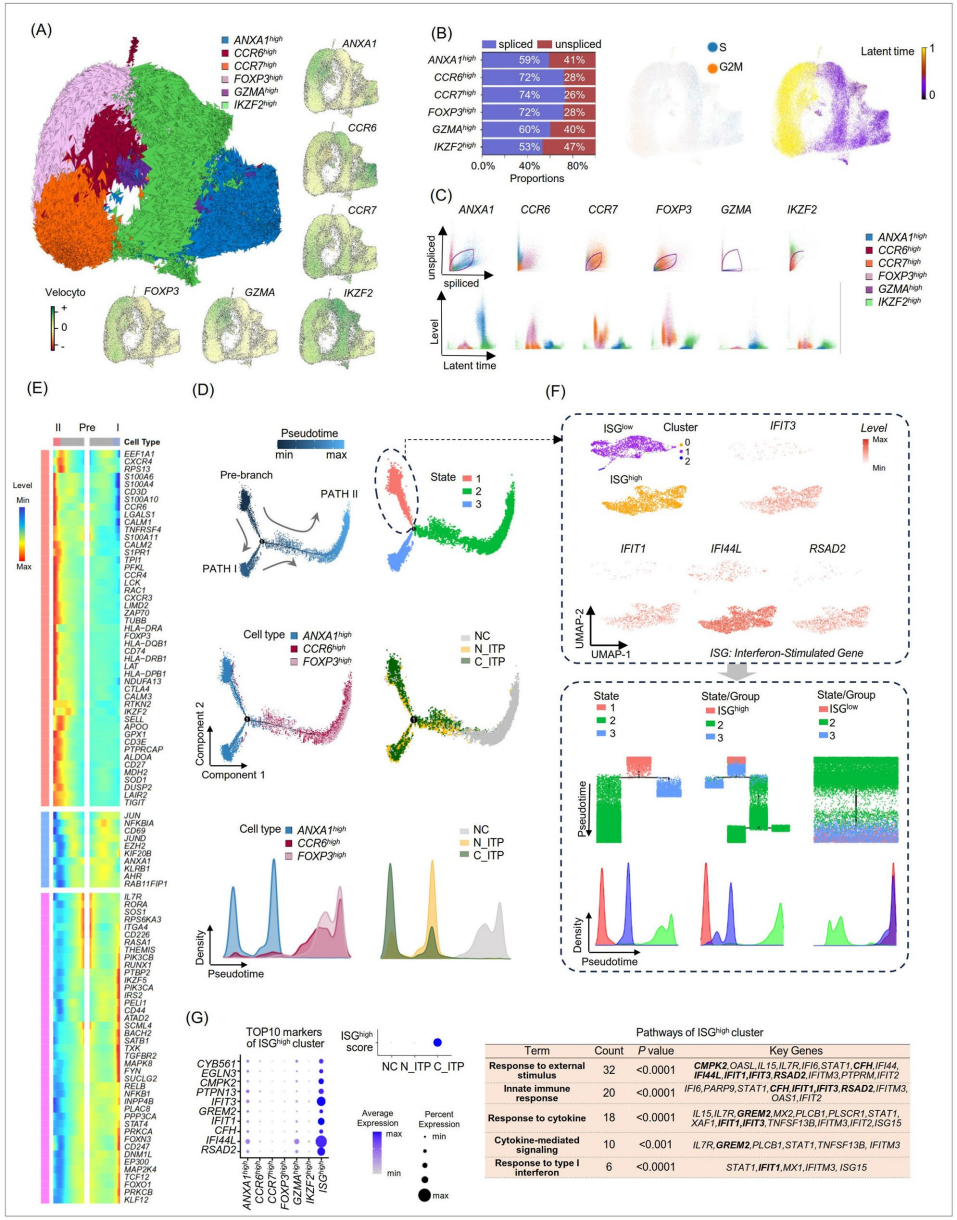
Dynamic transcriptomic and functional analysis of Treg subpopulations using single-cell Velocity (scVelo) and Monocle, with a focus on root cells. **A** Using the scVelo method, we analyzed the integrated scRNA-seq data of CD4^+^CD25^+^Tregs derived from NC, N_ITP, and C_ITP, highlighting the Velocyto of key genes such as *ANXA1*, *CCR6*, and *CCR7*. **B** The spliced and unspliced transcript ratios across different Treg subpopulations (left), cell cycle distribution (middle), and pseudotime trajectory analysis (right). **C** scVelo analysis of key genes and their expression dynamics along the pseudotime. **D-E** Pseudotime analysis using monocle for *ANXA1*^high^Treg, *CCR6*^high^Treg, and *FOXP3*^high^Treg (**D**), with heatmap visualization of significantly altered genes in pre-branch, path-I, and path-II (**E**). **F **Further Uniform Manifold Approximation and Projection (UMAP) clustering and annotation of the State 1 cells identified in (**D**), the expression levels of key genes were shown. Each subpopulation was extracted for more detailed pseudotime trajectory analysis. **G** Visualization of the top 10 gene expression levels in interferon-stimulated gene (ISG)^high^, scoring different Treg sources based on these genes, and conducting pathway enrichment analysis for genes significantly upregulated in the ISG^high^ cluster.

We next performed scVelo analysis on the integrated single-cell sequencing data of Tregs. Combined with the earlier results from Figs. 1C and 2A, we observed that the *IKZF2*^*high*^, *GZMA*^*high*^, and *CCR7*^*high*^ subpopulations exhibited high expression of their respective marker genes-*IKZF2*,* GZMA*, and *CCR7*. Concurrently, their RNA velocity also increased, indicating elevated transcriptional activity, and suggesting that these genes will remain highly expressed in their respective clusters in the future (Fig. 2A). Further analysis revealed that the dominant Treg subpopulations in ITP patients (*ANXA1*^*high*^, *IKZF2*^*high*^, *GZMA*^*high*^) showed an increased proportion of unspliced RNA, whereas the primary Treg subpopulations in normal individuals (*FOXP3*^*high*^, *CCR6*^*high*^, *CCR7*^*high*^) exhibited lower levels of unspliced RNA (Fig. 2B). This suggests that Tregs in ITP patients exhibit higher transcriptional activity. Moreover, we observed that the dominant Treg subpopulations in ITP patients were mainly in the S phase of the cell cycle, indicating ongoing DNA synthesis and proliferation, marking the initiation of differentiation (Fig. 2B). In contrast, Tregs from normal individuals were primarily in the G2/M phase, representing a later stage of differentiation (Fig. 2B). The proportions of spliced and unspliced RNA for key marker genes *ANXA1*,* CCR6*,* CCR7*,* FOXP3*,* GZMA*, and *IKZF2*, along with their transcriptional changes over pseudotime, are shown in Fig. 2C.

Interestingly, we found that while the *FOXP3*^*high*^ and *CCR6*^*high*^ clusters exhibited high expression of *FOXP3* and *CCR6* at the transcriptional level, their RNA velocity for *ANXA1* was also elevated, indicating a high potential for ANXA1 expression (Fig. 2A). Conversely, the *ANXA1*^*high*^ cluster displayed high RNA velocity for *CCR6*, suggesting a potential transcriptional transition between these clusters (Fig. 2A). Previous findings indicated that the *ANXA1*^*high*^ cluster is primarily derived from ITP patients, while the *FOXP3*^*high*^ and *CCR6*^*high*^ clusters are largely derived from normal individuals (Fig. 1B-C). This suggests a possible conversion between these Treg subpopulations, which may drive the transition of normal Tregs into ITP-associated Tregs, contributing to disease pathogenesis.

To explore this further, we extracted and merged the *ANXA1*^*high*^, *FOXP3*^*high*^, and *CCR6*^*high*^ clusters for Monocle analysis. The results revealed three differentiation states: State 1 cells were at the initiation of differentiation, State 3 represented intermediate stages, and State 2 corresponded to the terminal stages (Fig. 2D). Cluster-wise, *ANXA1*^*high*^ cells were distributed across the initiation and intermediate stages, while *CCR6* ^*high*^ and *FOXP3* ^*high*^ cells were found at the terminal stages (Fig. 2D). Source-wise, Tregs from C_ITP patients were located at the initiation stage, Tregs from N_ITP patients were at intermediate stages, and Tregs from NC were predominantly at the terminal stage (Fig. 2D). Notably, Tregs from N_ITP and C_ITP overlapped significantly with the *ANXA1*^*high*^ cluster along the differentiation trajectory, while the *FOXP3*^*high*^ and *CCR6*^*high*^ clusters overlapped with NC-derived Tregs (Fig. 2D). Consistent with this, naïve Treg markers scored significantly higher in C_ITP-derived Tregs (Fig. 1F), supporting the notion that these Tregs are immature and located at the initiation stage of differentiation, followed by N_ITP Tregs, with NC-derived Tregs being relatively more mature.

We further analyzed the expression dynamics of key immune genes during the Pre-branch (C_ITP) to PATH I (N_ITP Tregs) and PATH II (NC Tregs) differentiation trajectories. In Fig. 2E, *IL7R* expression appeared slightly higher in Pre-branch cells compared to terminal states. *IL7R* (*CD127*) is a key component of the interleukin-7 signaling pathway and is broadly expressed in naïve and memory T cells. In contrast, classical Tregs are typically characterized by low or absent CD127 expression, which is widely used for their identification. The modest expression of *IL7R* in pre-branch cells may suggest that these cells had not yet fully acquired the classical Treg phenotype. Heatmap analysis also showed that Pre-branch cells exhibited relatively high expression of enhancer of zeste 2 polycomb repressive complex 2 subunit (*EZH2*), *ANXA1*, and *KLRB1* (Fig. 2E). In PATH I, genes such as pellino E3 ubiquitin protein ligase 1 (*PELI1*), CD44, BTB domain and CNC homolog 2 (*BACH2*), SATB homeobox 1 (*SATB1*), transforming growth factor beta receptor 2 (*TGFBR2*), signal transducer and activator of transcription 4 (*STAT4*), transcription factor 12 (*TCF12*), and forkhead box O1 (*FOXO1*) were upregulated, while PATH II displayed progressive upregulation of immunosuppressive genes such as galectin 1 (*LGALS1*), *CCR6*,* CCR4*, LIM domain containing 2 (*LIMD2*), *FOXP3*,* CD74*, cytotoxic T-lymphocyte associated protein 4 (*CTLA4*), selectin L (*SELL*), and T cell immunoreceptor with Ig and ITIM domains (*TIGIT*) (Fig. 2E).

The above analysis suggests that State 1 is primarily composed of C_ITP-derived Tregs, State 2 consists mainly of NC-derived Tregs, and State 3 contains N_ITP-derived Tregs (Fig. 2D). To further investigate the factors maintaining the stability of C_ITP Tregs and the potential transition between ITP-derived and NC-derived Tregs, we performed UMAP clustering on State 1 cells (Fig. 2F). This revealed two subgroups: one with high expression of interferon-stimulated genes (ISGs, including interferon induced protein with tetratricopeptide repeats 3 (*IFIT3*), interferon induced protein with tetratricopeptide repeats 1 (*IFIT1*), interferon induced protein 44 like (*IFI44L*), and radical S-adenosyl methionine domain containing 2 (*RSAD2*)), designated as ISG^high^, and the other with low ISG expression, termed ISG^low^ (Fig. 2F). Monocle analysis of ISG^high^ and ISG^low^ groups combined with State 2 or State 3 cells revealed that ISG^high^ cells remained at the initial differentiation position, whereas ISG^low^ cells transitioned toward the terminal stage, overlapping with State 3 (Fig. 2F). This indicates that the ISG^high^ subgroup plays a critical role in maintaining the pathological features of C_ITP Tregs, while the ISG^low^ subgroup shares similar characteristics with N_ITP Tregs. Differential gene expression analysis between ISG^high^ and ISG^low^ subgroups identified the Top 10 upregulated genes in ISG^high^, including cytochrome b561(*CYB561*), *IFIT3*,* IFIT1*, and *IFI44L*, while ISG^low^ showed elevated expression of genes such as *KLRB1*, HOP homeobox (*HOPX*), ADAM metallopeptidase domain 12 (*ADAM12*), protein tyrosine phosphatase non-receptor type 13 (*PTPN13*), and phospholipase C beta 1 (*PLCB1*) (Fig. 2G, Figure S1). Scoring Tregs from each group based on these signature genes revealed that the ISG^high^ score was significantly elevated in C_ITP-derived Tregs but was minimal in the other groups (Fig. 2G). In contrast, the ISG^low^ score was low in NC-derived Tregs, moderately increased in N_ITP Tregs, and highest in C_ITP Tregs ( Figure S1). Finally, pathway enrichment analysis of genes upregulated in ISG^high^ cells showed enrichment in pathways such as response to external stimulus, innate immune response, response to cytokines, cytokine-mediated signaling, and response to type I interferon (Fig. 2G).

### *CCR6*^*high*^ and *FOXP3*^*high*^ marked the origin of NC-derived Tregs, while *ANXA1*^*high*^ initiated N_ITP and C_ITP Tregs, with ISG level altered across these immature-like Tregs

**Fig. 3:**
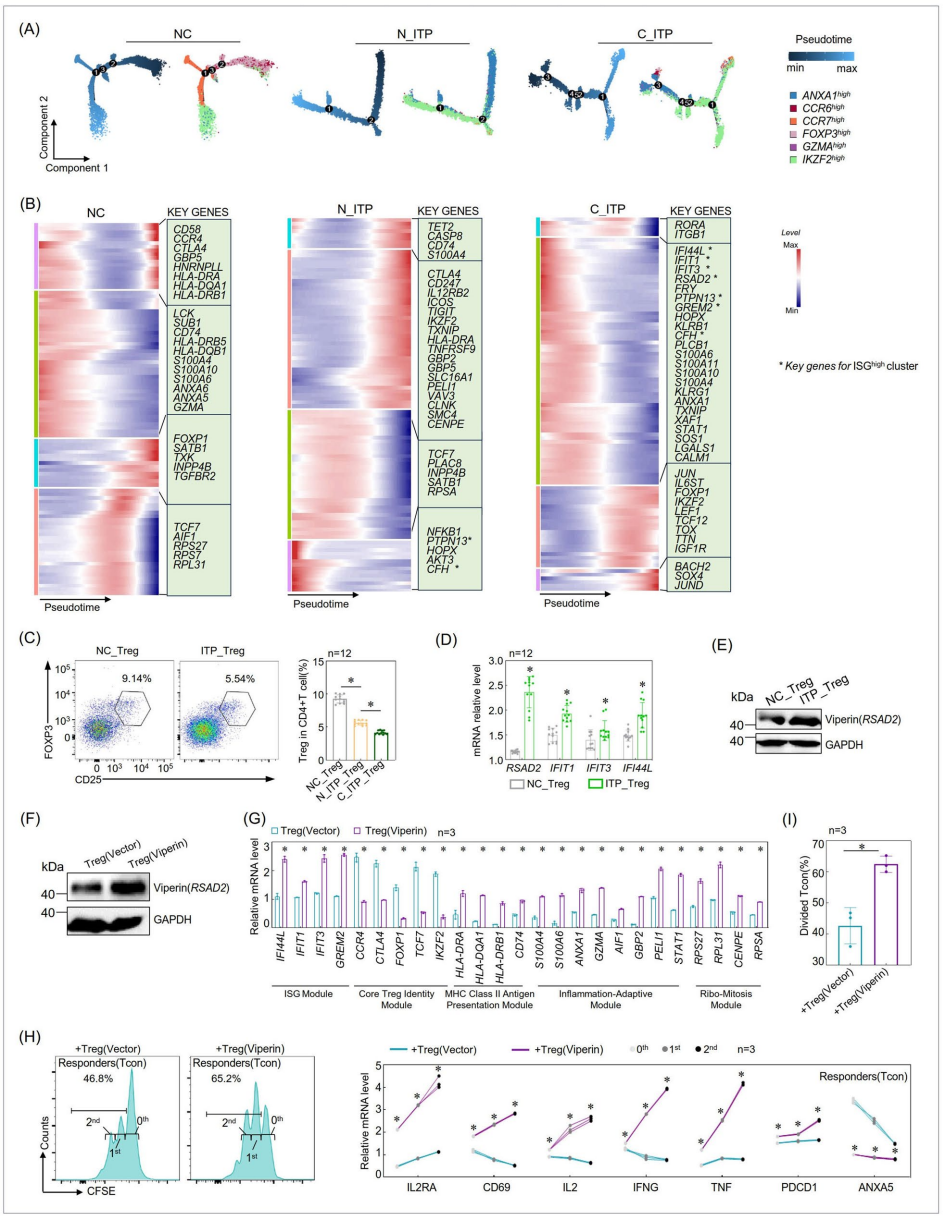
Pseudotime analysis of Tregs from different sources revealed dynamic changes in key genes such as ISGs. A critical ISG was identified, and its protein level and impact on Treg suppressive function were evaluated. **A** Monocle analysis of Tregs derived from NC, N_ITP, and C_ITP. **B** Pseudotime ordering of individual cell IDs from scRNA-seq of Tregs in each group, to observe the dynamic changes in key immune molecules along the pseudotime trajectory. **C**-**E** Analysis of the proportion of Tregs within CD4⁺ T cells from NC and ITP patients (**C**), comparison of ISGs’ transcript levels between the two groups (**D**), and comparison of Viperin protein levels (**E**). **F** Validation of Viperin overexpression in peripheral Tregs from normal individuals. **G** Changes in the expression of ISGs and key immunoregulatory molecules in Tregs following Viperin overexpression. **H**-**I** Treg suppression assays after Viperin overexpression, including analysis of *IL2RA*,* CD69*, and other gene transcription levels at different division cycles of Tcon cells (**H**), and statistical analysis of Treg-mediated suppression of Tcon proliferation (**I**). *P < 0.05; n, number; Tcon, conventional CD4⁺ T cell

To investigate how this differentiation trend manifests under distinct biological conditions, we separately analyzed Treg cells from NC, N_ITP, and C_ITP samples. While maintaining consistent cell annotations, we re-clustered the cells and reconstructed their pseudotime trajectories. As shown in Fig. 3A, *FOXP3*^*high*^ cells were positioned at the root of the trajectory in NC-derived Tregs, whereas *ANXA1*^*high*^ cells occupied the starting point in ITP-derived Tregs, suggesting that these two populations may serve as lineage precursors under physiological and pathological conditions, respectively. Combined with the predicted *ANXA1*^*high*^-to-*FOXP3*^*high*^ transition observed in Fig. 2, these results imply that Treg differentiation in ITP may be arrested at the *ANXA1*^*high*^ stage, thereby preventing progression into *FOXP3*^*high*^ cells and altering subsequent developmental trajectories.

To further characterize the differentiation programs, we examined the dynamic expression of immune-related genes along the pseudotime trajectories. Genes associated with immunosuppressive function, migration, and regulatory activity in Tregs exhibited distinct dynamic patterns between NC- and ITP-derived cells (Fig. 3B). Notably, at the beginning of the trajectories—corresponding to the *ANXA1*^*high*^ population–ISG-related genes were highly expressed in both N_ITP and C_ITP samples. Specifically, N_ITP-derived Tregs showed elevated expression of two representative ISG genes, including *PTPN13*, whereas C_ITP-derived Tregs exhibited high expression of six ISG markers, including *IFI44L*,* IFIT1*,* IFIT3*, and *RSAD2* (Fig. 3B). These findings are consistent with the previously highlighted enrichment of ISG^high^ cells within the *ANXA1*^*high*^ cluster (Fig. 2), further supporting the pathological significance of this population in the context of ITP.

### Upregulation of Viperin impaired the immunosuppressive function of Tregs in ITP

We analyzed peripheral blood-derived Treg cells from normal individuals and ITP patients and observed a significantly reduced frequency of Tregs in the peripheral blood of ITP patients compared to normal controls (Fig. 3C). Transcriptomic profiling of ISGs revealed markedly elevated expression of *RSAD2*,* IFIT1*,* IFIT3*,* and IFI44L* in ITP-derived Tregs, among which *RSAD2* exhibited the most pronounced upregulation (Fig. 3D). This observation was further validated at the protein level, where Viperin (*RSAD2*) expression was significantly higher in Tregs from ITP patients compared to those from the normal (Fig. 3E).

To investigate the functional implications of Viperin upregulation, we ectopically overexpressed Viperin in Tregs from normal individuals (Fig. 3F). Phenotypically, Viperin-overexpressing Tregs exhibited a marked shift away from a canonical suppressive identity, characterized by reduced expression of core immunosuppressive molecules and concomitant upregulation of stress response pathways, interferon-stimulated programs, antigen presentation machinery, and proliferation-related genes (Fig. 3G). These findings indicate that Viperin overexpression reprograms Tregs toward a dysfunctional, pro-inflammatory, and antigen-presenting-like phenotype, which may compromise peripheral immune tolerance.

We also examined cytokine expression at both the transcriptional and protein levels in Tregs derived from normal controls and ITP patients, as well as in Tregs before and after Viperin overexpression. The findings suggest that under autoimmune inflammatory conditions, Tregs from ITP patients may lose their phenotypic stability, as reflected by downregulation of the canonical suppressive cytokines IL-10 and TGF-β1 (Figure S4), leading to impaired immunosuppressive function. At the same time, inflammatory cues promoted aberrant secretion of effector cytokines IFN-γ, IL-17 A, and TNF (Figure S4), driving Tregs toward Th1- or Th17-like effector phenotypes. While type I interferons are known to induce expression of ISGs such as IFIT1 and IFIT3 [20–22], we did not detect altered expression of interferon alpha-2 (IFNA2) or interferon beta-1 (IFNB1) within Tregs from ITP patients (Figure S4A). Therefore, the elevated ISG expression (e.g., IFIT1 and IFIT3; Fig. 3D) observed in patient-derived Tregs is unlikely to result from intrinsic interferon production, but rather is most likely driven by extrinsic type I interferon signals.

Viperin (RSAD2) is a prototypical interferon-stimulated gene, and its overexpression resembles a “viral infection-like” state [23–29]. Maybe through molecular interactions and metabolic stress [23–29], Viperin overexpression led to upregulation of ISGs such as IFIT1 and IFIT3 (Fig. 3G), while simultaneously disrupting the Treg regulatory network. This resulted in reduced expression of IL-10 and TGF-β1 (Figure S4) and aberrant production of IFN-γ, IL-17 A, and TNF (Figure S4), conferring an effector-like inflammatory phenotype. Collectively, Tregs overexpressing Viperin displayed a phenotype consistent with that observed in ITP patient-derived Tregs, indicating that Viperin may act as a key regulator of Treg instability in ITP and may represent a potential therapeutic target.

Functional suppression assays confirmed that Viperin overexpressing Tregs exhibited significantly attenuated suppressive capacity (Fig. 3H-I). Co-cultured Tcon cells displayed earlier onset and sustained activation, as evidenced by upregulation of *IL2RA* (CD25) and *CD69* from the first division cycle (generation 0), with persistent expression across subsequent divisions, reflecting robust proliferative and activation programs (Fig. 3H). In this context, *IL-2* production was markedly elevated (Fig. 3H), facilitating autocrine expansion and amplifying Tcon responses. Additionally, *IFNG* and *TNF* were strongly upregulated in the Viperin overexpressing group, indicating enhanced Th1 polarization (Fig. 3H). Although programmed cell death protein 1 (*PDCD1*) was expressed under both conditions (Fig. 3H), its co-expression with *IFNG* in the Viperin overexpressing group suggests that Tcon cells were not functionally suppressed but instead remained in a state of chronic activation.

When Tregs overexpressing Viperin were co-cultured with Tcon cells, annexin A5 (ANXA5) levels in Tcon cells showed only a slight decrease with increasing division number, with a flatter decline compared to the control group, and ANXA5 mRNA level also remained consistently lower than that in controls (Fig. 3H). Viability assays further demonstrated that Tcon cells co-cultured with Viperin-overexpressing Tregs exhibited overall higher survival than those in the control group, with a modest increase in viability across successive divisions that was less pronounced than in controls (Figure S5). This phenomenon may be explained by the intact suppressive function of control Tregs, which highly express CD25 and strongly compete for IL-2, thereby promoting IL-2 deprivation–induced activation-induced cell death (AICD) in Tcon cells and resulting in elevated ANXA5. With increasing division numbers, more resilient Tcon populations are selectively maintained, leading to a gradual reduction in ANXA5 levels. In contrast, Viperin-overexpressing Tregs display impaired suppressive activity and reduced interleukin-2 (IL-2) consumption, thereby lowering the incidence of AICD in Tcon cells. Consequently, ANXA5 levels are overall reduced, and due to the diminished initial apoptotic pressure, the decline across division cycles appears comparatively flatter.

Taken together, these results demonstrate that Viperin overexpression impairs the regulatory function of Tregs, resulting in dysregulated Tcon activation, Th1-skewing, pro-inflammatory cytokine production, and apoptosis resistance (Fig. 3H-I). These findings provide mechanistic insight into how aberrant Viperin expression in Tregs may contribute to the disruption of immune tolerance and the pathogenesis of ITP.

### Distinct driver genes and transcriptional regulatory states in Treg subpopulations highlight aberrant signaling in ITP-derived Tregs

Using scVelo analysis, we identified the top five driver genes in each Treg subpopulation. In the *ANXA1*^*high*^ cluster, driver genes included *IFI44L*,* ANXA1*, and *KLRB1*; in the *CCR6*^*high*^ cluster, driver genes included protein tyrosine phosphatase receptor type C associated protein (*PTPRCAP*); in the *CCR7*^*high*^ cluster, driver genes included *CCR7*; in the *FOXP3*^*high*^ cluster, driver genes included *RTKN2*; in the *GZMA*^*high*^ cluster, driver genes included *IFI44L*; and in the *IKZF2*^*high*^ cluster, driver genes included *PELI1* (Figure S2A). Expression analysis of these genes revealed distinct transcriptional profiles: in NC-derived Tregs (primarily clustered in *FOXP3*^*high*^, *CCR6*^*high*^, and *CCR7*^*high*^ subpopulations), genes such as *CCR7*,* PTPRCAP*,* LIMD2*, ribosomal protein L30 (*RPL30*), and eukaryotic translation elongation factor 1 alpha 1 (*EEF1A1*) showed significantly increased transcriptional levels (Figure S2A). In N_ITP-derived Tregs, genes such as *PELI1*, signal transducing adaptor molecule (*STAM*), rhotekin 2 (*RTKN2*), and JunD proto-oncogene, AP-1 transcription factor subunit (*JUND*) were significantly upregulated, while C_ITP-derived Tregs showed elevated transcription of *ANXA1* and *IFI44L* (Figure S2A). These findings align with our previous analyses.

**Fig. 4:**
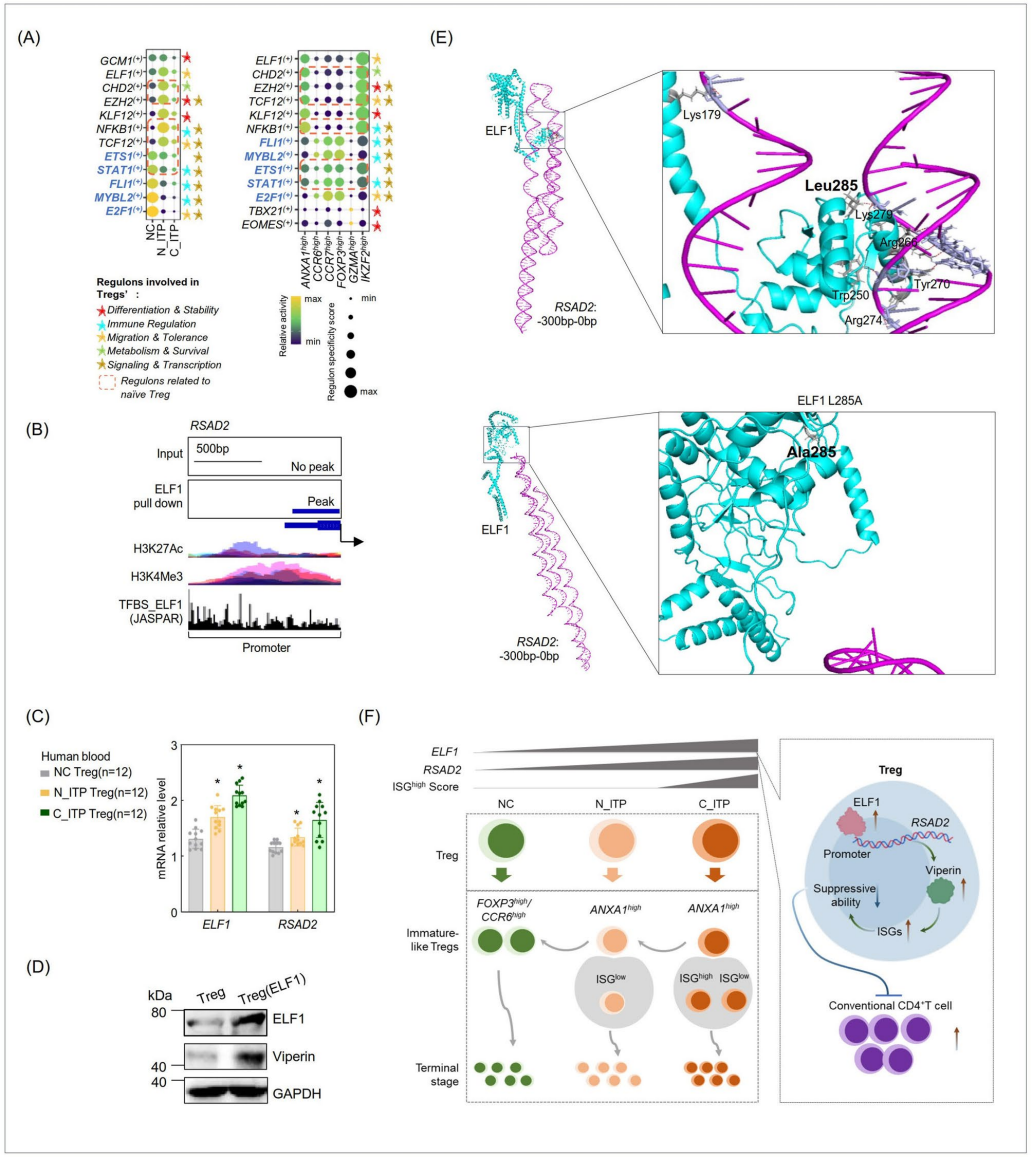
Transcription factor ELF1 regulates Viperin expression in Tregs and schematic overview of the study. **A** Regulon activity analysis of TFs across Treg subsets using pySCENIC. **B** ChIP-seq analysis of ELF1 binding to the *RSAD2* promoter region, along with histone modification profiles obtained via UCSC Genome Browser (https://genome.ucsc.edu/). Transcription factor binding sites (TFBS) were identified and annotated using the JASPAR database. **C** Validation of *ELF1* and *RSAD2* transcriptional levels in peripheral Tregs from NC and ITP patients at different disease stages. **D** Western blot analysis of Viperin protein expression following ELF1 overexpression in Tregs. **E** Prediction of ELF1 binding sites within the − 300 bp to 0 bp region upstream of the *RSAD2* gene using AlphaFold3 and PyMOL; mutational analysis of key amino acid residues was performed to assess ELF1-*RSAD2* interaction. **F** Schematic summary of the proposed mechanism investigated in this study, the right section was drawn by BioGDP(https://biogdp.com/).*P<0.05

Next, we performed pySCENIC analysis, which identified differentially active TF regulons between NC-derived and ITP-derived Tregs. In NC-derived Tregs (primarily located in *FOXP3*^*high*^, *CCR6*^*high*^, and *CCR7*^*high*^ subpopulations), regulons with increased activity included ETS proto-oncogene 1, transcription factor (*ETS1*)^(+)^, signal transducer and activator of transcription 1 (*STAT1*)^(+)^, Fli-1 proto-oncogene, ETS transcription factor (*FLI1*)^(+)^, MYB proto-oncogene like 2 (*MYBL2*)^(+)^, and E2F transcription factor 1 (*E2F1*)^(+)^ (Fig. 4A). Conversely, ITP-derived Tregs exhibited significant upregulation of regulons such as E74 like ETS transcription factor 1 (*ELF1*)^(+)^, chromodomain helicase DNA binding protein 2 (*CHD2*)^(+)^, *EZH2*^(+)^, KLF transcription factor 12 (*KLF12*)^(+)^, nuclear factor kappa B subunit 1 (*NFKB1*)^(+)^, and *TCF12*^(+)^ (Fig. 4A). These findings indicate distinct transcriptional states between normal and pathological Tregs.

Using STRING analysis, we predicted regulatory interactions between the TFs identified in Fig. 4A and the aforementioned molecules, creating a regulatory network map (Figure S2B). Further, we detailed each TF’s regulon, their target genes, and the associated motifs (Figure S2C). Comprehensive analysis revealed that key TFs in NC-derived Tregs include *STAT1*,* E2F1*, and *FLI1*, which are critical for maintaining normal Treg immune regulatory pathways, MHC-related processes, immunosuppressive functions, and cell migration (Figure S2B). In N_ITP-derived Tregs, TFs such as *EZH2* and *ELF1* play pivotal roles in driving pathological Treg signaling dysregulation, altering the cell cycle, and promoting proliferation. C_ITP-derived Tregs exhibited marked activation of transcription factors *EZH2*,* NFKB1*, and *ELF1* ( Figure S2B). Those findings reflect extensive transcriptional reprogramming and functional dysregulation in ITP-associated Tregs.

### Transcript factor ELF1 facilitated Viperin expression in Tregs

To elucidate the upstream transcriptional regulation of *RSAD2* in Tregs, we analyzed ChIP-seq datasets to assess the binding potential of TFs that were upregulated in ITP-derived Tregs (as shown in Fig. 4A) at the *RSAD2* locus. Among the candidate TFs, only ELF1 exhibited a distinct binding peak within the promoter region of *RSAD2*, suggesting a direct regulatory role (Fig. 4B). This region also showed increased H3K4Me3 enrichment, consistent with a transcriptionally active chromatin state (Fig. 4B). In addition, multiple ELF1 binding motifs were predicted within the − 300 to 0 bp upstream region of the *RSAD2* promoter (Fig. 4B). We further examined the expression levels of *ELF1* and *RSAD2* in clinical Treg samples. Both genes showed a progressive and statistically significant increase from NC-derived Tregs, to N_ITP-derived Tregs, and C_ITP-derived Tregs (Fig. 4C), supporting a potential *ELF1-RSAD2* regulatory axis associated with disease progression. Overexpression of ELF1 in Tregs significantly upregulated Viperin protein levels (Fig. 4D). Furtherly, we examined the genes regulated by RSAD2 as shown in Fig. 3G. We found that the expression changes of these genes upon ELF1 overexpression were consistent with those observed following RSAD2 overexpression (Fig. 3G&S3), supporting our conclusion that ELF1 positively regulates RSAD2.

To further explore the molecular mechanism of ELF1-mediated transcriptional activation, we analyzed the *RSAD2* promoter region and identified Leucine at position 285 (Leu285) of ELF1 as a putative key residue responsible for binding to the − 300 to 0 bp region (Fig. 4E). Collectively, these findings revealed a mechanistic link by which ELF1 directly regulated RSAD2 transcription in Tregs, contributing to their altered phenotype in the context of ITP.

## Discussion

ITP is an autoimmune disorder with an incompletely understood pathogenesis [1, 2]. Tregs are pivotal immune-suppressive cells that play a critical role in maintaining immune homeostasis and preventing the development of autoimmune diseases by suppressing excessive immune responses [3, 4]. It has been well-established that Treg dysfunction or a reduction in their numbers can trigger severe autoimmune disorders [4, 30]. For ITP patients receiving low-dose decitabine, the number and function of Treg cells were significantly improved compared to pre-treatment levels, while Th1 and Th17 cells were suppressed, leading to a restoration of immune balance [6, 7]. However, the lack of effective therapies targeting Tregs in ITP is primarily due to the unclear mechanisms underlying Treg dysfunction in this disease. To address this, we provide a novel single-cell analysis to comprehensively investigate the heterogeneity of Tregs in ITP patients and explore the dynamic changes in Treg function and key characteristics as individuals progress from normal status to N_ITP, and further into C_ITP. Our analysis revealed distinct differences between Tregs derived from ITP patients and those from NC, with even further heterogeneity observed between Tregs from N_ITP and C_ITP patients (Fig. 1D). In ITP, the proportion of Tregs expressing *ANXA1*^*high*^, *IKZF2*^*high*^, and *GZMA*^*high*^ was markedly increased, and these subpopulations became the dominant fraction within the overall Treg pool (Figs. 1B-C&E). Tregs from ITP patients displayed immune functional impairments, including elevated naïve Treg markers (Figs. 1F-G), suggesting that as normal Tregs transition into pathological Tregs in ITP, their core features and the predominant cellular populations undergo significant alterations.

ANXA1 is a calcium-dependent membrane-binding protein from the Annexin family, known for its role in regulating cell membrane stability, immune responses, and inflammation [12]. ANXA1 exerts its effects through interactions with phospholipids and calcium ions and has been shown to possess anti-inflammatory properties, particularly in conditions of excessive inflammation [13, 31]. Studies suggest that ANXA1 can induce the generation of M2-like macrophages and microglia through its receptor, thereby promoting a Treg-driven immunosuppressive tumor microenvironment [17]. Furthermore, ANXA1 has been implicated in enhancing Treg function and is associated with poor survival in breast cancer patients [10]. Targeting ANXA1 has been shown to diminish Treg function and reduce tumor size in breast cancer models [10]. FOXP3 is the central TF that sustains the stability of Tregs, regulating a range of genes critical for Treg-mediated immune suppression [16]. CCR6 and CCR7 are chemokine receptors involved in Treg migration, enabling their trafficking to sites of immune response, which is essential for Treg function and the maintenance of immune homeostasis [3, 11, 32]. IKZF2, a member of the Ikaros family of TFs, enhances Treg immune function and serves as a marker of naïve Tregs [3, 14]. GZMA, expressed in natural killer cells and cytotoxic T cells, is also found in activated Tregs [33, 34]. Through the secretion of GZMA, Tregs induce target cell apoptosis [15]. Our findings reveal that *ANXA1*^*high*^, *FOXP3*^*high*^, and *CCR6*^*high*^ Tregs are predominantly located at the initiation stage of differentiation in ITP and NC-derived Tregs, respectively (Fig. 3A). Further analysis of these populations demonstrated that the *ANXA1*^*high*^ cluster, predominantly derived from C_ITP patients, is positioned at the early differentiation stage and exhibits progenitor-like characteristics (Fig. 2). The *ANXA1*^*high*^ cluster from N_ITP patients represents an intermediate differentiation stage, whereas *FOXP3*^*high*^ and *CCR6*^*high*^ Tregs, primarily derived from NC, are located at the terminal stage of differentiation (Fig. 2). Tregs from C_ITP patients displayed primitive and undifferentiated features, characterized by high transcriptional activity (Fig. 2), which may account for their reduced immunosuppressive capacity. In contrast, Tregs from N_ITP patients represent an intermediate differentiation state, while NC-derived Tregs exhibit markers typically associated with fully functional, immunosuppressive Tregs, indicative of a more mature Treg phenotype (Fig. 2).

ISGs are a group of genes that are upregulated in response to interferon signaling, playing a pivotal role in antiviral immunity, cell survival, and immune regulation [35, 36]. ISGs are crucial in T cell activation, proliferation, and immune responses, particularly in promoting IFN-γ production [36–38]. In Tregs, the core molecule maintaining immune suppression is FOXP3 [3, 18]. However, when Tregs produce effector molecules such as IFN-γ, they lose their immunosuppressive function [5, 18]. Our analysis showed that as the ISG^low^ score increases, Tregs derived from NCs gradually transition to N_ITP and ultimately to C_ITP-derived Tregs (Figure S1). Notably, Tregs from C_ITP patients exhibited a pathological shift, characterized by a significantly elevated ISG^high^ score (Fig. 2). The maintenance of C_ITP-derived Tregs, particularly the ANXA1^high^ subpopulation, was largely driven by the ISG^high^ subpopulation (Fig. 2). These results suggest that, in terms of immature-like Tregs, NC-derived Tregs maintain their core mature functionality, whereas N_ITP-derived Tregs gradually lose their distinctive Treg features, exhibiting lower ISG levels. In contrast, C_ITP-derived Tregs exhibit elevated ISG scores and have undergone a qualitative shift towards an effector-like phenotype, losing their original Treg characteristics. This shift could enable the further differentiation of these Tregs into downstream populations, establishing distinct Treg pools.

RSAD2, an ISG known for encoding the antiviral protein viperin [39], was a potential pathogenic factor. RSAD2 has been implicated in various autoimmune and inflammatory conditions [39]. It has been shown to contribute to pregnancy complications in systemic lupus erythematosus (SLE) by promoting placental lipid accumulation and impairing vascular development, an effect alleviated by the RSAD2 inhibitor L-chicoric acid [40]. Additionally, RSAD2 mediates the RSAD2–ISG15 axis in mature dendritic cells and is associated with activation of NLR signaling in endothelial cells [41]. Silencing RSAD2 in B cells from patients with primary Sjögren’s syndrome attenuates their hyperactivation via suppression of the NF-κB pathway, suggesting its potential as a therapeutic target [42]. Our results revealed a marked upregulation of Viperin in Treg cells from ITP patients; overexpression of Viperin impaired the suppressive function of Tregs (Fig. 3E-I), indicating that Viperin may activate pro-inflammatory signaling pathways in Tregs, ultimately compromising their regulatory capacity. Furthermore, we identified ELF1 as a key transcription factor driving Viperin expression in Tregs (Figure 4). ELF1 has been previously reported as a potential biomarker for SLE [43], with established roles in disease susceptibility [44] and reduced TCR zeta chain expression in SLE patients [45].To our knowledge, this is the first study to demonstrate that ELF1 contributes to Treg dysfunction in ITP by transcriptionally activating RSAD2, providing new mechanistic insights into the dysregulation of Treg-mediated immune tolerance in autoimmune disease.

As summarized in Fig. 4F, this study provides a comprehensive characterization of the phenotypic heterogeneity and functional reprogramming of Tregs in ITP. Tregs derived from ITP patients displayed distinct molecular and functional signatures compared to normal controls, with dynamic alterations in key markers such as *ANXA1*,* FOXP3*, and *CCR6* during disease progression. The transition from normal to dysfunctional Tregs was marked by a loss of immunosuppressive capacity and a shift toward an effector-like phenotype, particularly in C_ITP, where ISG activity was markedly upregulated. Among these ISGs, RSAD2(Viperin) emerged as a critical mediator of Treg dysfunction. We demonstrated that the transcription factor ELF1 promoted RSAD2(Viperin) expression, thereby impairing Treg suppressive function. These findings highlight *ANXA1*, Viperin, and ELF1 as potential therapeutic targets for restoring Treg-mediated immune regulation in ITP. Collectively, our study advances the understanding of Treg dysregulation in ITP and provides a mechanistic framework for the development of targeted immunotherapies aimed at re-establishing immune tolerance in autoimmune diseases.

## Supplementary Information

Supplementary Material 1.

Supplementary Material 2.

Supplementary Material 3.

Supplementary Material 4.

## Data Availability

No datasets were generated or analysed during the current study.

## Abbreviations

ITP: Primary immune thrombocytopenia
Tregs: Regulatory T cells
ISG: Interferon-stimulated gene
scRNA-seq: Single-cell RNA sequencing
NC: Normal control
N_ITP: Newly diagnosed ITP
C_ITP: Chronic ITP
RT-qPCR: Real-time reverse transcription quantitative polymerase chain reaction
FACS: Fluorescence-activated cell sorting
UMAP: Uniform manifold approximation and projection
ChIP-seq: Chromatin immunoprecipitation sequencing
IGV: Integrative genomics viewer
PCA: Principal component analysis
GSVA: Gene set variation analysis
GO: Gene ontology
scVelo: Single-cell velocity
k-NN: k-nearest neighbor
pySCENIC: python implementation of Single-Cell rEgulatory Network Inference and Clustering
TF: Transcription factor
ELISA: Enzyme-linked immunosorbent assay
FOXP3: Forkhead box P3
IFN-γ: Interferon gamma
TGF-β1: Transforming growth factor beta 1
IL-10: Interleukin-10
TNF-α: Tumor necrosis factor alpha
IL-17A: Interleukin-17A
TMB, 3,3′,5,5′: tetramethylbenzidine
ANXA1: Annexin A1
CCR: C-C Motif Chemokine Receptor
IKZF2: Ikaros Family Zinc Finger 2
GZMA: Granzyme A
TCR: T-cell receptor
FAO: Fatty acid oxidation
NKR: Natural killer receptor
KLRB1: Killer cell lectin like receptor B1
TCA: Tricarboxylic acid
IFN: Interferon
IL7R: Interleukin 7 receptor
EZH2: Enhancer of zeste 2 polycomb repressive complex 2 subunit
PELI1: Pellino E3 ubiquitin protein ligase 1
BACH2: BTB domain and CNC homolog 2
SATB1: SATB homeobox 1
TGFBR2: Transforming growth factor beta receptor 2
STAT4: Signal transducer and activator of transcription 4
TCF12: Transcription factor 12
FOXO1: Forkhead box O1
LGALS1: Galectin 1
LIMD2: LIM domain containing 2
CTLA4: Cytotoxic T-lymphocyte associated protein 4
SELL: Selectin L
TIGIT: T cell immunoreceptor with Ig and ITIM domains
IFIT3: Interferon induced protein with tetratricopeptide repeats 3
IFIT1: Interferon induced protein with tetratricopeptide repeats 1
IFI44L: Interferon induced protein 44 like
RSAD2: Radical S-adenosyl methionine domain containing 2
CYB561: Cytochrome b561
HOPX: HOP homeobox
ADAM12: ADAM metallopeptidase domain 12
PTPN13: Protein tyrosine phosphatase non-receptor type 13
PLCB1: Phospholipase C beta 1
IFNA2: Interferon alpha-2
IFNB1: Interferon beta-1
PD1: Programmed cell death protein 1
ANXA5: Annexin A5
AICD: Activation-induced cell death
IL-2: Interleukin-2
PTPRCAP: Protein tyrosine phosphatase receptor type C associated protein
RTKN2: Rhotekin 2
RPL30: Ribosomal protein L30
EEF1A1: Eukaryotic translation elongation factor 1 alpha 1
STAM: Signal transducing adaptor molecule
RTKN2: Rhotekin 2
JUND: JunD proto-oncogene, AP-1 transcription factor subunit
ETS1: ETS proto-oncogene 1, transcription factor
STAT1: Signal transducer and activator of transcription 1
FLI1: Fli-1 proto-oncogene, ETS transcription factor
MYBL2: MYB proto-oncogene like 2
E2F1: E2F transcription factor 1
ELF1: E74 like ETS transcription factor 1
CHD2: Chromodomain helicase DNA binding protein 2
KLF12: KLF transcription factor 12
NFKB1: Nuclear factor kappa B subunit 1
SLE: Systemic lupus erythematosus
