# Supplementary figures and images for: Interferon-stimulated Viperin impairs Treg function in autoimmune thrombocytopenia
Source: Cell Commun Signal. 2025 Nov 20;23:500. doi: 10.1186/s12964-025-02511-6 (PMC12632067; doi:10.1186/s12964-025-02511-6)

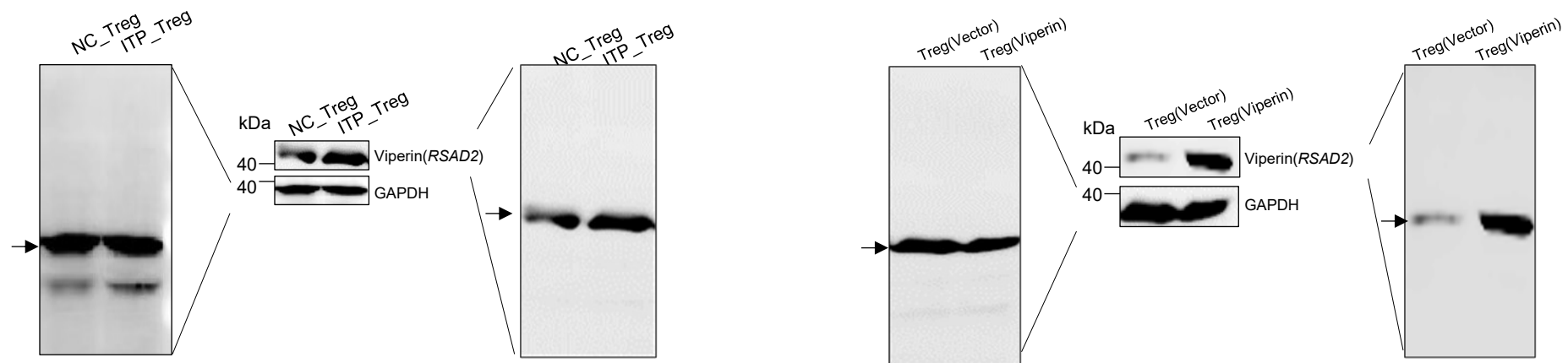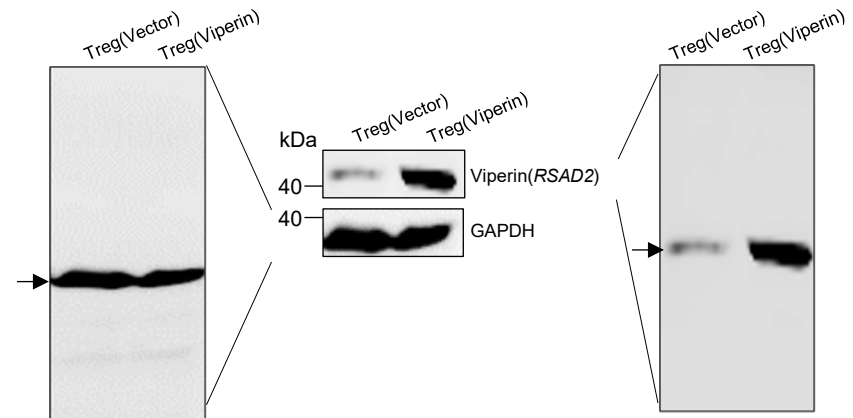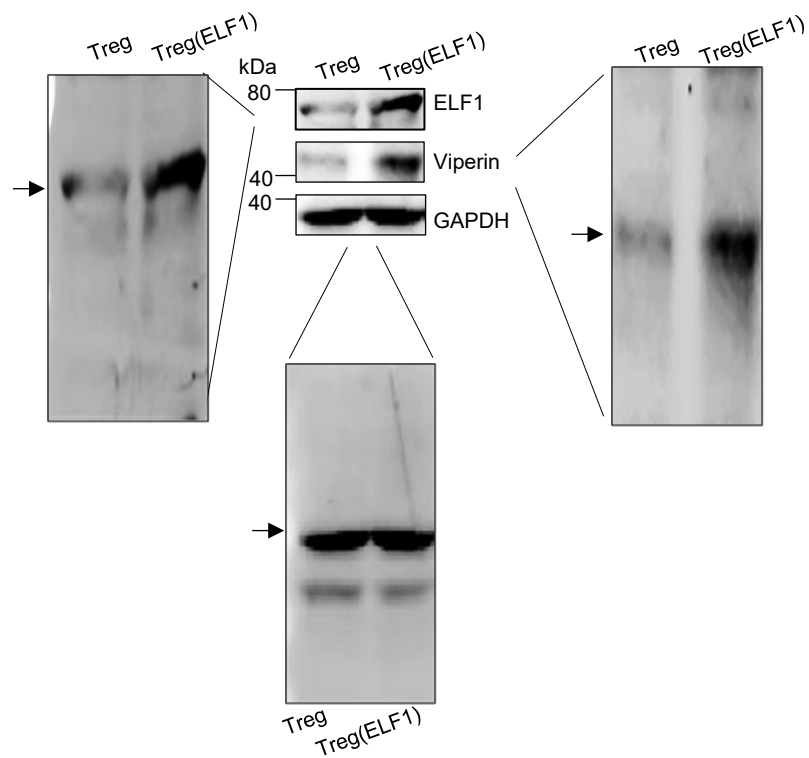

Supplement: Supplementary file 1 — Supplementary Material 1. [file 12964_2025_2511_MOESM1_ESM.pdf]
