# Supplementary material for: Interferon-stimulated Viperin impairs Treg function in autoimmune thrombocytopenia
Source: Cell Commun Signal. 2025 Nov 20;23:500. doi: 10.1186/s12964-025-02511-6 (PMC12632067; doi:10.1186/s12964-025-02511-6)
Supplement: Supplementary file 2 — Supplementary Material 2. [file 12964_2025_2511_MOESM2_ESM.pptx]

## Slide 1
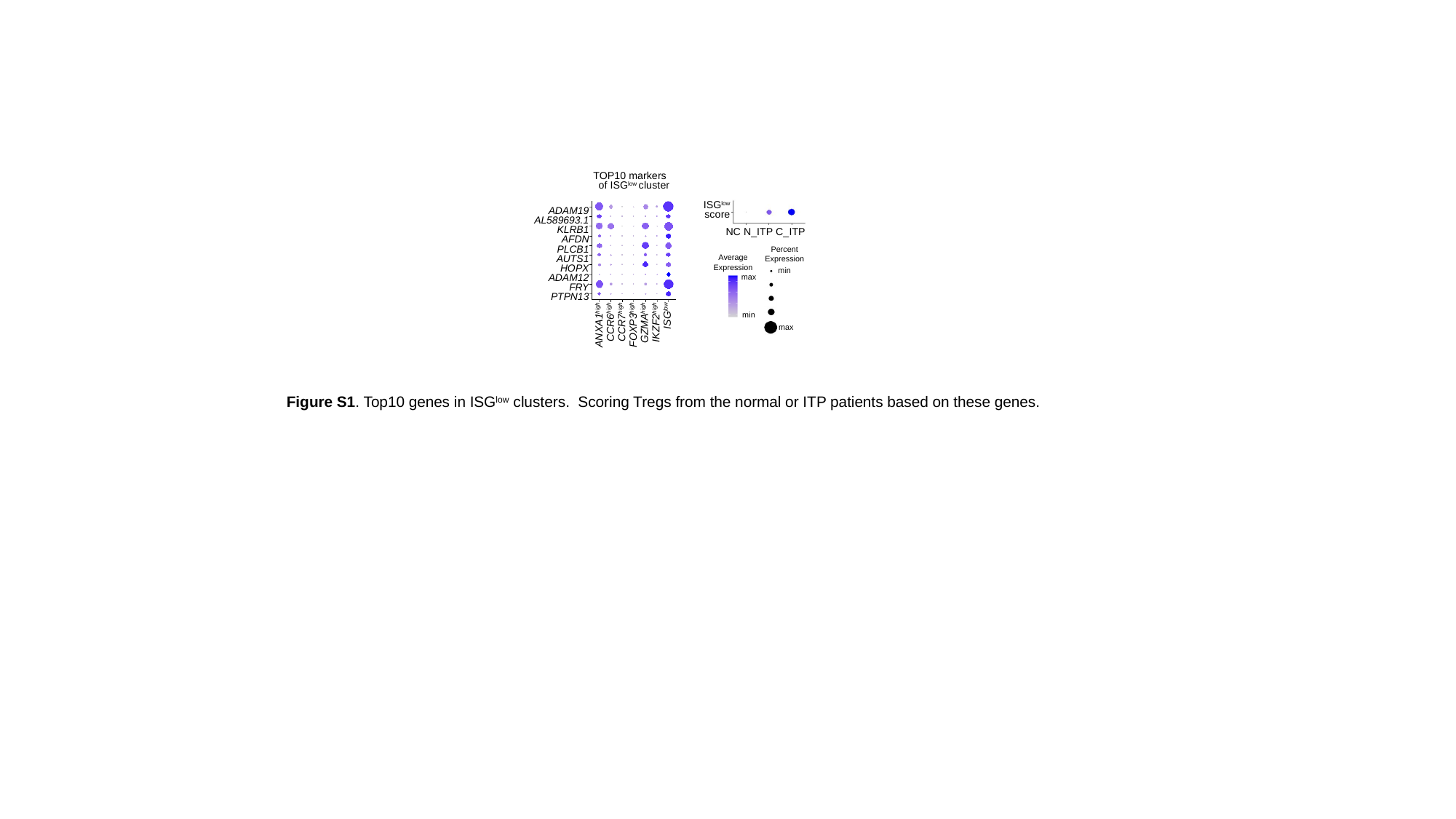

TOP10 markers
of ISGlow cluster
ADAM19
AL589693.1
KLRB1
AFDN
PLCB1
AUTS1
HOPX
ADAM12
FRY
PTPN13
ANXA1high
CCR6high
CCR7high
FOXP3high
GZMAhigh
IKZF2high
ISGlow
ISGlow score
NC N_ITP C_ITP
Percent Expression
min
max
Average Expression
max
min
Figure S1. Top10 genes in ISGlow clusters. Scoring Tregs from the normal or ITP patients based on these genes.

## Slide 2
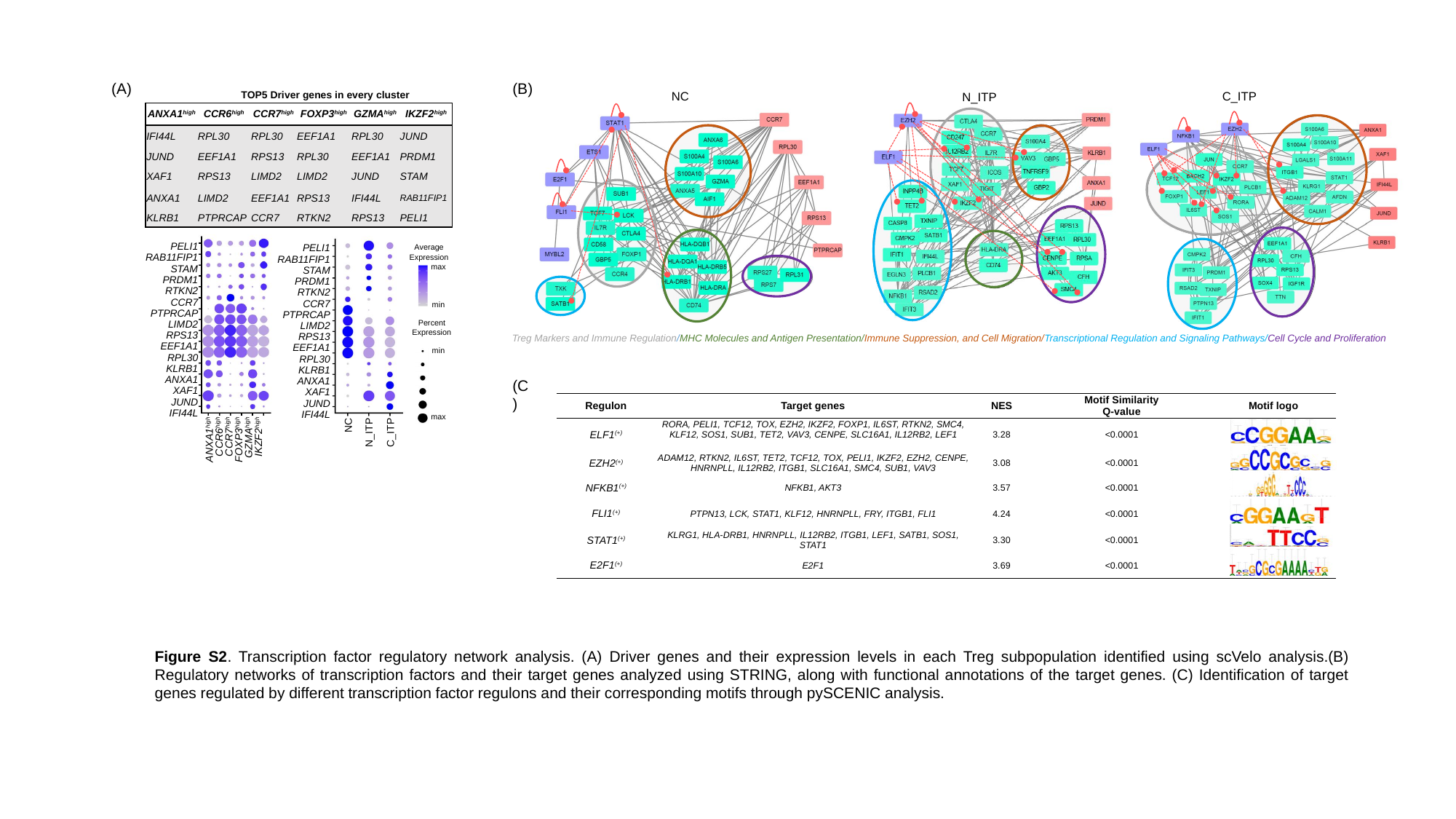

(B)
(A)
TOP5 Driver genes in every cluster
| ANXA1high | CCR6high | CCR7high | FOXP3high | GZMAhigh | IKZF2high |
| --- | --- | --- | --- | --- | --- |
| IFI44L | RPL30 | RPL30 | EEF1A1 | RPL30 | JUND |
| JUND | EEF1A1 | RPS13 | RPL30 | EEF1A1 | PRDM1 |
| XAF1 | RPS13 | LIMD2 | LIMD2 | JUND | STAM |
| ANXA1 | LIMD2 | EEF1A1 | RPS13 | IFI44L | RAB11FIP1 |
| KLRB1 | PTPRCAP | CCR7 | RTKN2 | RPS13 | PELI1 |
PELI1
RAB11FIP1
STAM
PRDM1
RTKN2
CCR7
PTPRCAP
LIMD2
RPS13
EEF1A1
RPL30
KLRB1
ANXA1
XAF1
JUND
IFI44L
Average Expression
PELI1
RAB11FIP1
STAM
PRDM1
RTKN2
CCR7
PTPRCAP
LIMD2
RPS13
EEF1A1
RPL30
KLRB1
ANXA1
XAF1
JUND
IFI44L
max
min
Percent Expression
min
ANXA1high
CCR6high
CCR7high
FOXP3high
GZMAhigh
IKZF2high
max
NC
N_ITP
C_ITP
NC
C_ITP
N_ITP
Treg Markers and Immune Regulation/MHC Molecules and Antigen Presentation/Immune Suppression, and Cell Migration/Transcriptional Regulation and Signaling Pathways/Cell Cycle and Proliferation
(C)
| Regulon | Target genes | NES | Motif Similarity Q-value | Motif logo |
| --- | --- | --- | --- | --- |
| ELF1(+) | RORA, PELI1, TCF12, TOX, EZH2, IKZF2, FOXP1, IL6ST, RTKN2, SMC4, KLF12, SOS1, SUB1, TET2, VAV3, CENPE, SLC16A1, IL12RB2, LEF1 | 3.28 | <0.0001 | |
| EZH2(+) | ADAM12, RTKN2, IL6ST, TET2, TCF12, TOX, PELI1, IKZF2, EZH2, CENPE, HNRNPLL, IL12RB2, ITGB1, SLC16A1, SMC4, SUB1, VAV3 | 3.08 | <0.0001 | |
| NFKB1(+) | NFKB1, AKT3 | 3.57 | <0.0001 | |
| FLI1(+) | PTPN13, LCK, STAT1, KLF12, HNRNPLL, FRY, ITGB1, FLI1 | 4.24 | <0.0001 | |
| STAT1(+) | KLRG1, HLA-DRB1, HNRNPLL, IL12RB2, ITGB1, LEF1, SATB1, SOS1, STAT1 | 3.30 | <0.0001 | |
| E2F1(+) | E2F1 | 3.69 | <0.0001 | |
Figure S2. Transcription factor regulatory network analysis. (A) Driver genes and their expression levels in each Treg subpopulation identified using scVelo analysis.(B) Regulatory networks of transcription factors and their target genes analyzed using STRING, along with functional annotations of the target genes. (C) Identification of target genes regulated by different transcription factor regulons and their corresponding motifs through pySCENIC analysis.

## Slide 3
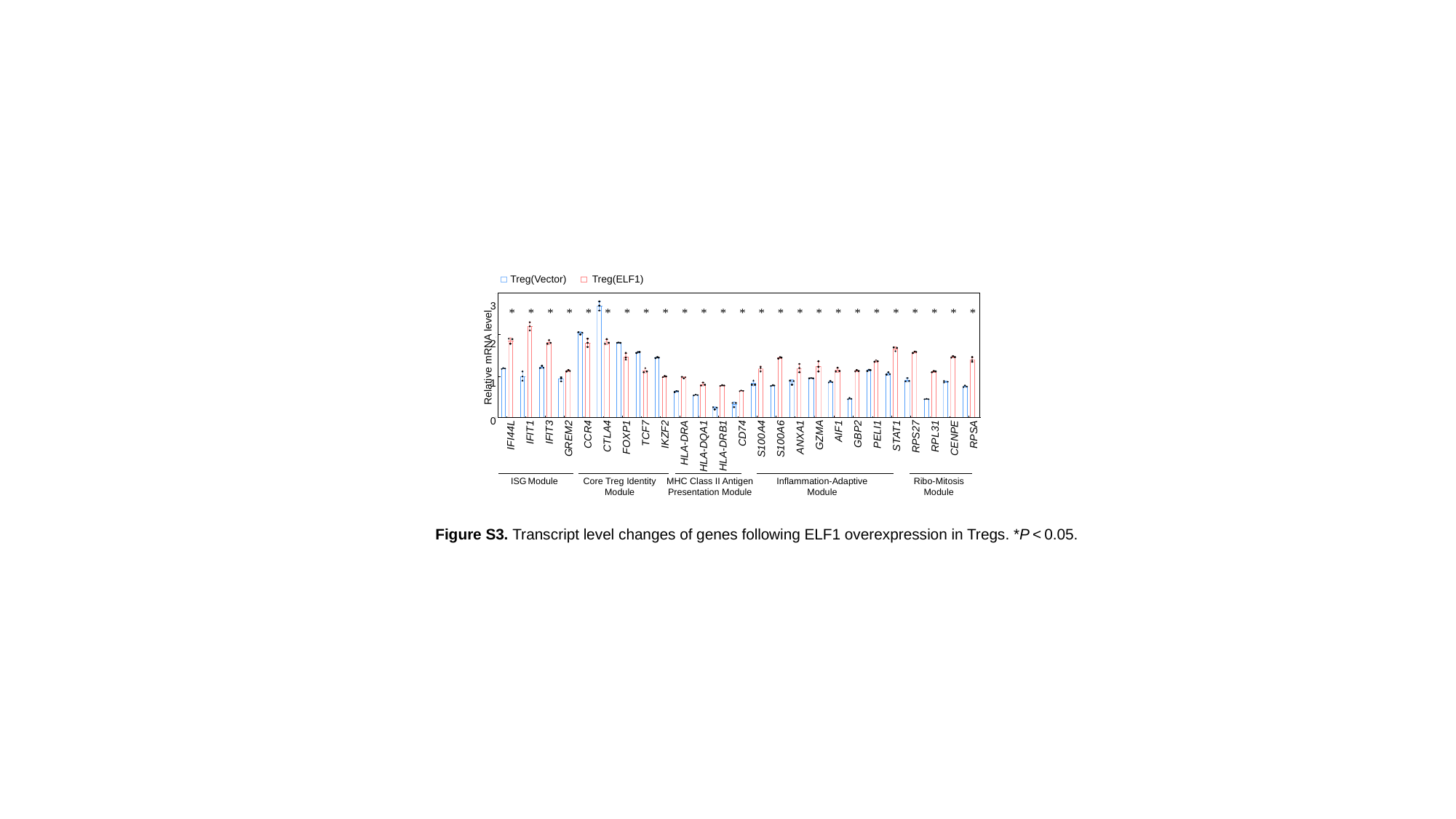

IFI44L
IFIT1
IFIT3
GREM2
CCR4
CTLA4
FOXP1
TCF7
IKZF2
HLA-DRA
HLA-DQA1
HLA-DRB1
CD74
S100A4
S100A6
ANXA1
GZMA
AIF1
GBP2
PELI1
STAT1
RPS27
RPL31
CENPE
RPSA
3
2
1
0
Treg(Vector)
Treg(ELF1)
*
*
*
*
*
*
*
*
*
*
*
*
*
*
*
*
*
*
*
*
*
*
*
*
*
Relative mRNA level
ISG Module
Core Treg Identity Module
MHC Class II Antigen Presentation Module
Inflammation-Adaptive Module
Ribo-Mitosis Module
Figure S3. Transcript level changes of genes following ELF1 overexpression in Tregs. *P < 0.05.

## Slide 4
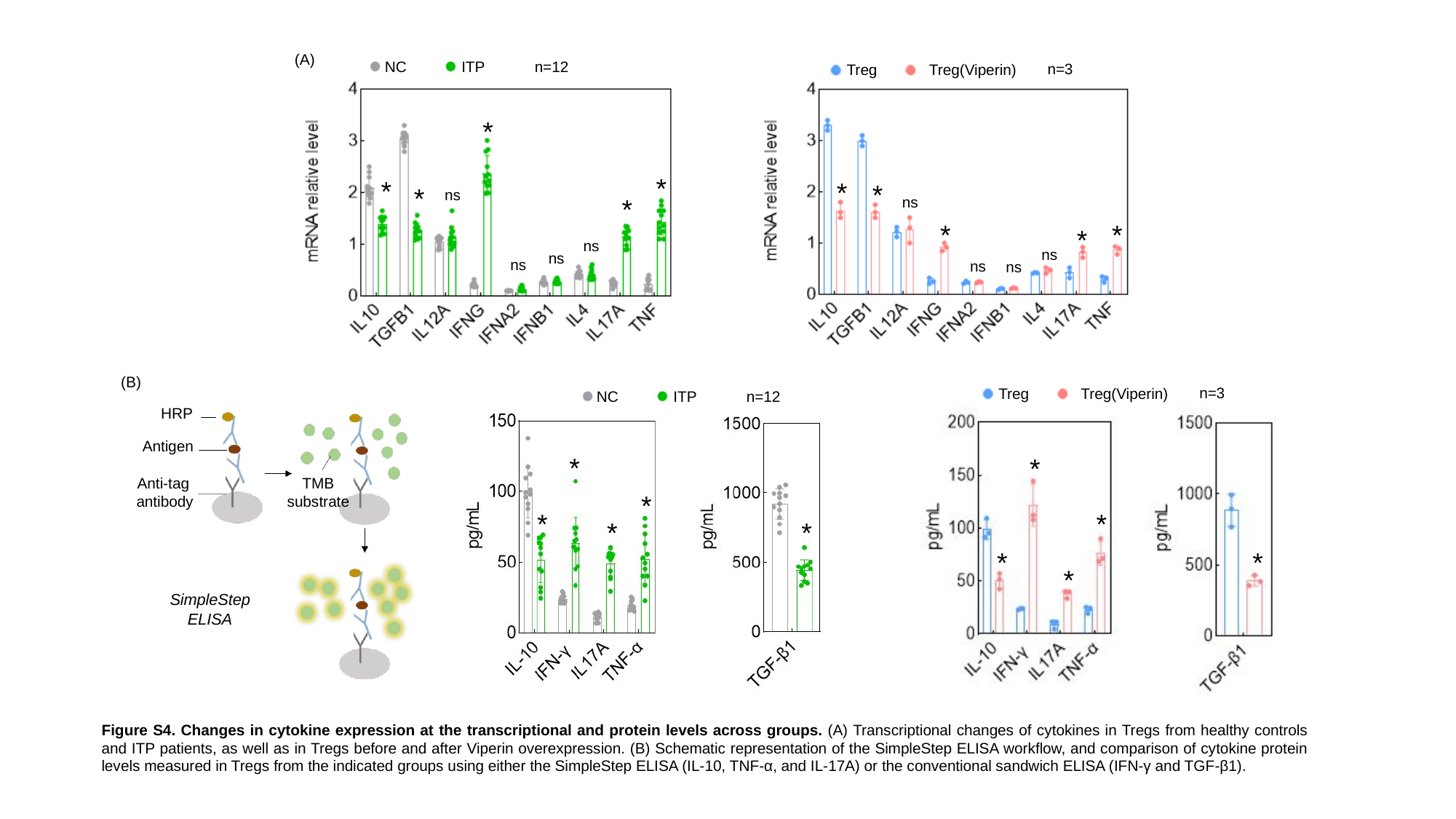

(A)
NC
ITP
n=12
n=3
Treg
Treg(Viperin)
*
*
*
*
*
*
ns
ns
*
*
*
*
ns
ns
ns
ns
ns
ns
(B)
n=3
Treg
Treg(Viperin)
NC
ITP
n=12
HRP
*
*
*
*
*
*
*
*
*
*
Antigen
Anti-tag
antibody
TMB substrate
SimpleStep ELISA
Figure S4. Changes in cytokine expression at the transcriptional and protein levels across groups. (A) Transcriptional changes of cytokines in Tregs from healthy controls and ITP patients, as well as in Tregs before and after Viperin overexpression. (B) Schematic representation of the SimpleStep ELISA workflow, and comparison of cytokine protein levels measured in Tregs from the indicated groups using either the SimpleStep ELISA (IL-10, TNF-α, and IL-17A) or the conventional sandwich ELISA (IFN-γ and TGF-β1).

## Slide 5
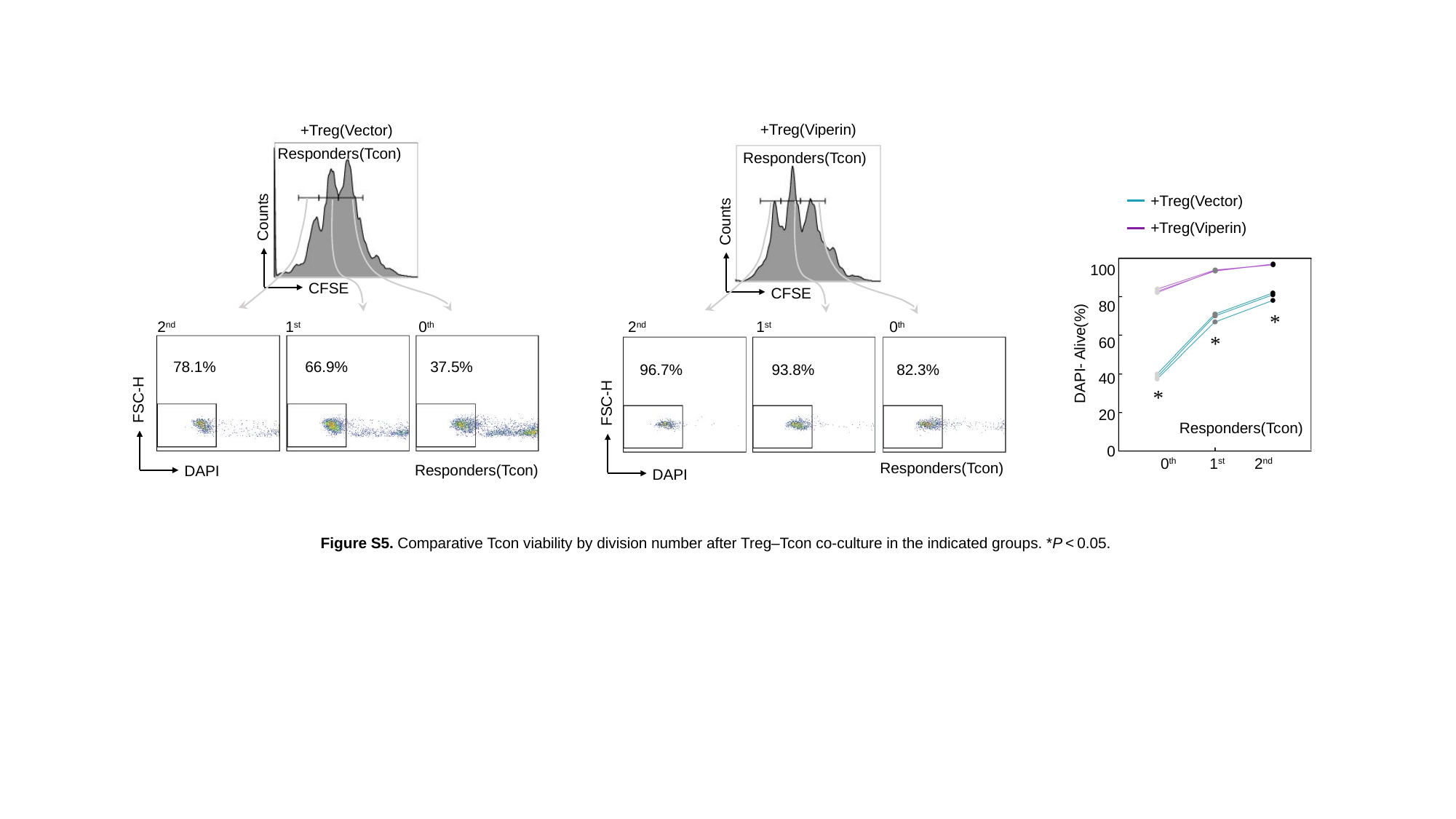

+Treg(Viperin)
Responders(Tcon)
Counts
CFSE
2nd
1st
0th
96.7%
93.8%
82.3%
FSC-H
Responders(Tcon)
DAPI
+Treg(Vector)
Responders(Tcon)
Counts
CFSE
2nd
1st
0th
78.1%
66.9%
37.5%
FSC-H
Responders(Tcon)
DAPI
+Treg(Vector)
+Treg(Viperin)
100
80
60
40
20
0
*
*
DAPI- Alive(%)
*
Responders(Tcon)
0th 1st 2nd
Figure S5. Comparative Tcon viability by division number after Treg–Tcon co-culture in the indicated groups. *P < 0.05.
